# Pupil Sizes Scale with Attentional Load and Task Experience in a Multiple Object Tracking Task

**DOI:** 10.1371/journal.pone.0168087

**Published:** 2016-12-15

**Authors:** Basil Wahn, Daniel P. Ferris, W. David Hairston, Peter König

**Affiliations:** 1 Institute of Cognitive Science, University of Osnabrück, Osnabrück, Germany; 2 Human Neuromechanics Laboratory, School of Kinesiology, University of Michigan – Ann Arbor, MI, United States of America; 3 Human Research and Engineering Directorate, U.S. Army Research Laboratory, Aberdeen, MD, United States of America; 4 Department of Neurophysiology and Pathophysiology, Center of Experimental Medicine, University Medical Center Hamburg-Eppendorf, Hamburg, Germany; Monash University, AUSTRALIA

## Abstract

Previous studies have related changes in attentional load to pupil size modulations. However, studies relating changes in attentional load and task experience on a finer scale to pupil size modulations are scarce. Here, we investigated how these changes affect pupil sizes. To manipulate attentional load, participants covertly tracked between zero and five objects among several randomly moving objects on a computer screen. To investigate effects of task experience, the experiment was conducted on three consecutive days. We found that pupil sizes increased with each increment in attentional load. Across days, we found systematic pupil size reductions. We compared the model fit for predicting pupil size modulations using attentional load, task experience, and task performance as predictors. We found that a model which included attentional load and task experience as predictors had the best model fit while adding performance as a predictor to this model reduced the overall model fit. Overall, results suggest that pupillometry provides a viable metric for precisely assessing attentional load and task experience in visuospatial tasks.

## Introduction

Typically, pupil size changes are linked to changes in the amount of light that reaches the retina (a reflex known as the “pupil light reflex” [1, 2]) or whether a close or distant object is viewed (a response known as the “pupil near response” [1, 3]). However, for about 120 years, pupil sizes have been found to be additionally and systematically influenced by cognitive processing [3, 4]. For instance, pupil size changes have been studied in decision-making [5–8], attention [4, 9–22], emotions [23, 24], language [25], and memory [26–29].

A structure in the brain that is functionally involved in the control of pupil size changes is the locus coeruleus [16, 30–33]. The locus coeruleus projects to a wide range of brain regions but primarily to regions related to attentional processing such as the parietal cortex and the superior colliculus [34, 35], suggesting that pupil sizes are influenced by attentional processing. Relatedly, pupil size changes often have been associated with attentional processing when task difficulties were varied [9, 16, 18, 36–38]. For instance, when performing arithmetic tasks of varying difficulty, pupil sizes increase with increasing task difficulty [36–38] and these findings also generalize to other attention demanding tasks [9, 16, 18]. However, studies that have investigated increases in attentional processing over a larger spectrum of difficulty levels and on a finer scale are scarce. In fact, attentional demands typically range along two levels (e.g., [9, 18]).

A task that has been suggested to be ideal to study increases in attentional processing over a larger spectrum of difficulty levels [18, 39, 40] and associated physiological correlates [41] is the so-called multiple object tracking (“MOT”) task [42]. In this task, a subset of several objects are indicated as “targets” at the beginning of a trial on a computer screen. Then, objects become indistinguishable and move randomly across the screen. After several seconds, objects stop moving and participants need to indicate which objects were the targets. In a recent study, Alnæs and colleagues [16] investigated how modulations in pupil sizes systematically changed as a function of attentional load in a MOT task (i.e., the number of targets that needed to be tracked). In particular, they varied the number of targets between two and five objects and also included a passive viewing condition in which participants did not track any targets. They found that pupil sizes increased with each increase in attentional load, suggesting that pupil size modulations are related to task difficulty also over a larger spectrum of attentional load manipulations. The stability of the pupil size modulations by the attentional load in the MOT task was assessed by correlating the modulations in a first session with modulations in several subsequent recording sessions that were recorded within the span of several years. They found a high correlation between sessions, suggesting that pupil size modulations related to attentional load are stable over a long time. Overall, the study by Alnæs and colleagues [16] found a close relationship between pupil sizes and attentional load in a MOT task that is stable over a long time.

These insights [16], however, raise a number of new questions. An important aspect that has not been addressed is the influence of task experience on pupil size modulations. In particular, it was not investigated how training effects over several sessions within a few days can modulate pupil sizes. Repeated performance of tasks such as a MOT task leads to improved performance. Thus, the repeated execution of a MOT task could lead to a dissociation between attentional load and task difficulty (i.e., the attentional load manipulation remains constant while the task difficulty decreases with task experience). We hypothesized that pupil sizes could systematically decrease with increasing task experience as participants adapt to the task requirements and devote less processing resources to the task. Such a finding would demonstrate that pupil size modulations are also affected by task experience and not merely by the attentional load manipulations in the MOT task.

Another important question is how performing a task as such and increasing the attentional load in a task affect pupil sizes. In the study by Alnæs and colleagues [16], a condition in which participants tracked only one target was not included (i.e., tracking load was only varied between two and five targets, and a passive viewing condition was included). However, including a one target condition does allow to compare pupil size modulations relative to the passive viewing condition (i.e., when no MOT task is performed and the moving objects are passively viewed). We hypothesized that this comparison involves two factors that influence pupil size modulations: 1) a change in attentional load (i.e., tracking one target more) and 2) the switch in attention demands from performing no task to performing a task. For the remaining comparisons involving conditions in which targets are tracked (e.g., between tracking one and two targets), we hypothesized that only the former factor (i.e., the increase of attentional load) would affect pupil sizes. Therefore, we predicted that the magnitude of the pupil size modulations between performing no task in comparison to performing a task should be larger than the pupil size modulations due to performing a task at a lower compared to a higher task difficulty level. As a point of note, in both these cases the attentional load manipulation is the same (i.e., the number of tracked targets is increased by one).

In addition, given all these partially redundant factors that could influence pupil size modulations (i.e., attentional load, task performance, and task experience), we address the question of which combination of this redundant set of predictors leads to the highest explanatory power (i.e., model fit) for predicting pupil size modulations.

Taken together, we address how changes in attentional load that span the complete spectrum of load manipulations (i.e., from no load manipulation up to a condition of high attentional load) systematically affect pupil sizes. Moreover, we address to what extent attentional load, task experience and/or performance influence pupil sizes.

## Results

**Fig 1:**
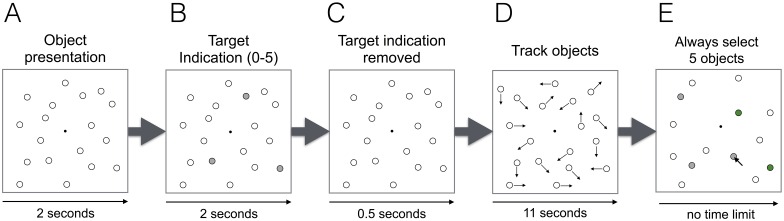
Multiple object tracking (MOT) task trial overview. (A) Objects (total: 18) are shown. (B) A subset of objects (i.e., the “targets”, varied between zero and five) turn gray for two seconds. Note that in the passive viewing condition (zero targets), no objects turn gray in this phase. (C) The target indication is removed. Targets are now indistinguishable from the other objects. (D) Objects randomly move across the screen and participants need to track the movements of the targets. Arrows indicate the current movement direction of the objects. (E) The objects stop moving and participants select the targets using the mouse. To keep motor demands constant, participants always need to select five objects but only the first selections count to performance (e.g., if two targets need to be tracked, then only the first two selections count to performance). After five objects are selected, feedback is given (i.e., correct selections turn green).

We investigated 20 participants’ (14 female, *M* = 23.03 years, *SD* = 3.34 years) performance in a MOT task at varying levels of attentional load (Fig 1). In particular, the number of targets to be tracked varied from zero, which served as a passive viewing condition, up to five targets. This allowed us to investigate whether pupil size changes between a passive viewing condition and tracking one target are different from changes in pupil sizes between conditions in which targets are tracked. Moreover, to investigate effects of task experience, each participant performed the experiment on three consecutive days.

**Fig 2:**
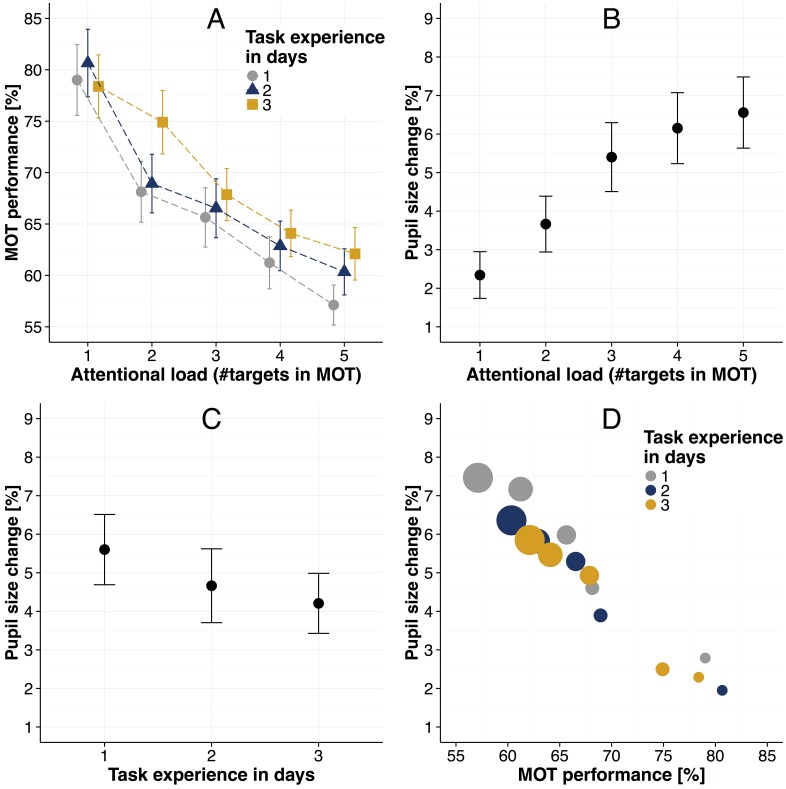
Main experiment results. (A) MOT task performance (i.e., fraction of correctly selected targets) as a function of attentional load and task experience. (B) Pupil size changes relative to the passive viewing condition as a function of attentional load. (C) Pupil size changes relative to the passive viewing condition as a function of task experience. (D) MOT task performance plotted against the pupil size changes, separately for each day. In addition, the number of targets in the MOT task is coded by the sizes of the circles (i.e., with increasing circle size, the number of targets increases). Error bars in all panels are standard error of the mean.

An overview of the MOT task performance (i.e., fraction of correctly selected targets) is shown in Fig 2A. Performance systematically decreased with an increasing number of targets in the MOT task. Moreover, participants slightly improved their tracking performance over days. We tested whether these observations were statistically reliable using linear mixed models and model comparisons [43]. For this purpose, we first constructed a baseline model, in which only intercepts were fitted as a random effect (i.e., intercepts were fitted for each participant individually to account for the repeated measures design [44]). Then, we subsequently added predictors to the baseline model and assessed via model comparisons whether these added predictors significantly explain additional variance in the data. As a model selection criterion, we report the Bayesian Information Criterion (“BIC”) for each addition. The BIC is a measure of how well a model fits data that is corrected for the number of included predictors. We first added attentional load (i.e., the number of targets tracked in the MOT task) to the model as a fixed and random effect (i.e., slopes are fitted for each participant individually) and found that these additions significantly explained additional variance in the data (*χ*^2^(3) = 170.31, *p* < .001, BIC = 2195.5—for comparison, the baseline model’s BIC = 2348.7). We then added task experience (i.e., the index of day of measurement) as a fixed and random effect to the model and found that these additions significantly explain additional variance in the data (*χ*^2^(4) = 38.13, *p* < .001, BIC = 2180.2). Adding an interaction effect between attentional load and task experience did not significantly explain additional variance in the data (*χ*^2^(1) = 1.17, *p* = .280, BIC = 2184.8).

For the final model, in which only attentional load and task experience were included, we assessed the regression coefficients and their significance. We found a significant negative slope (*B* = -4.67, SE = 0.62, *p* < .001) for attentional load, suggesting that participants’ performance on average decreased with increasing number of targets by about five percent for each added target. The regression coefficient for task experience was not significant (*B* = 1.62, SE = 0.98, *p* = .114). However, as noted above, using a more sensitive test, the model comparisons when adding task experience to the model yielded a significant comparison, suggesting that task experience does explain variance in task performance in addition to the variance explained by attentional load.

In sum, participants’ performance in the MOT task decreased with increasing attentional load and we found a slight improvement in performance with increasing task experience. These findings indicate that the experimental manipulation of attentional load (i.e., varying the number of tracked targets) was successful in having the intended effect as task performance systematically decreased with increasing attentional load.

As a next step, we investigated pupil sizes (Fig 2B, 2C and 2D) and their relation to attentional load, task performance and task experience. We averaged over the median pupil sizes of the left and right eye and normalized the resulting values relative to the passive viewing condition (for more details see methods section “Methods of data analysis”). Note that although the total moving time amounted to eleven seconds, pupil size data were only extracted between three and nine seconds in which the objects were moving (a similar time window was also used in [16]). This circumvents potential perceptual effects like contrast changes during target indication and executive effects like motor preparation to select targets after objects’ motion in the data. In addition, when inspecting pupil size traces across the whole eleven seconds (see S1 Fig), pupil size differences between attentional load conditions appear to develop within the first seconds of a trial and then remain relatively constant throughout the rest of the trial.

Pupil sizes systematically increased with attentional load (Fig 2B) and decreased with task experience (Fig 2C). When relating pupil sizes to the MOT performance, a highly negative relation can be observed (i.e., with increasing performance, pupil sizes decrease) (Fig 2D). Summarizing, on a descriptive level, the data suggests that pupil size modulations depend on the attentional load, task experience, and task performance. Moreover, these observations suggest that this set of variables is partially redundant in explaining variance in the pupil size modulations.

**Fig 3:**
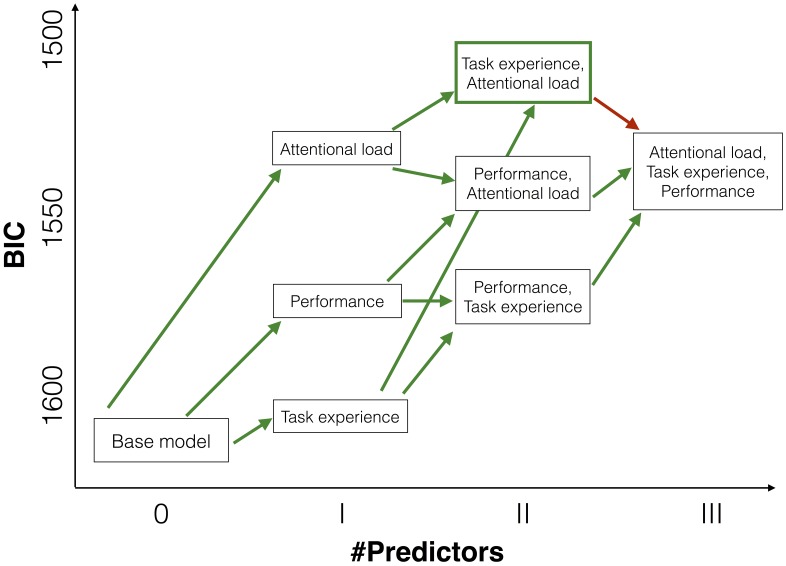
Model comparisons overview. The ordinate shows the model fit in form of the reported Bayesian information criterion (BIC). The abscissa indicates the number of predictors in the model. Green arrows indicate model additions that significantly explained additional variance to the variance explained by previous predictors. Red arrows indicate that the added predictors did not explain additional variance.

On an inferential level, to investigate which are the most relevant variables in the redundant set (i.e., attentional load, task experience, and task performance), we used linear mixed models and model comparisons in close analogy to the investigation of MOT task performance above. In the baseline model, we fitted intercepts for each participant individually to account for the repeated measurements design. We then added attentional load as a fixed and random effect to the model. We found that in comparison to the baseline model (BIC = 1611.1, Fig 3 box “Base model”) these additions significantly explained more variance in the data (*χ*^2^(3) = 96.65, *p* < .001, BIC = 1531.6, Fig 3 box “Attentional load”). We then added task experience as an additional fixed and random effect to the model and found that these additions were significant as well (*χ*^2^(4)=40.59, *p* < .001, BIC = 1513.8, Fig 3 box “Task experience, Attentional load”). As a point of note, when inspecting the pupil size traces across the tracking period for each day (see S1 Fig), the effect of task experience appears to be driven by a greater reduction of pupil sizes (over days) after an initial increase in pupil sizes in the first few seconds of a trial. Adding an interaction effect between task experience and attentional load as a fixed and random effect to the model did not lead to a significant increase in explained variance (*χ*^2^(5)=5.25, *p* = .386, BIC = 1537.0), suggesting that there is no interaction effect between task experience and attentional load.

In principle, attentional load and task experience might already sufficiently predict changes in the pupil sizes while performance in the MOT task does not explain substantial additional variance. Conversely, it could be that the task performance in the MOT task explains significant additional variance in the pupil size data and replaces none, one, or both of the other variables. To investigate this question, we extended the modeling approach described above. We used attentional load, task experience, and task performance as predictors in all possible combinations to predict pupil size modulations. Then, we computed whether adding a variable does significantly explain more variance in the data (Fig 3). Notably, for the model that had attentional load and task experience as predictors, we found that adding performance as an additional predictor did not explain additional variance in the data (*χ*^2^(5)=8.16, *p* = .150, BIC = 1534.2, see red arrow in Fig 3). Thus, the complete model including all three predictors was not the best. Yet, the performance could significantly explain additional variance if it was added to a model in which only the predictor attentional load (*χ*^2^(4) = 14.10, *p* = .007, BIC = 1540.3) or task experience (*χ*^2^(4)=63.89, *p* < .001, BIC = 1567.1) was included. Moreover, of all possible models, when comparing the BICs, a model considered above with attentional load and task experience, but not including task performance was preferred (BIC = 1513.8). Overall, we conclude that task performance did not explain enough additional variance on top of the variance explained by attentional load and task experience in the pupil size data in order to justify its inclusion. Moreover, given the BICs for all tested models, a model including only attentional load and task experience as predictors is preferred.

We then assessed the regression coefficients and their significance for the model that provided the best BIC (i.e., the model including attentional load and task experience as predictors). We found a significant positive slope for attentional load (*B* = 1.09, *SE* = 0.13, *p* < .001), suggesting that participants’ pupil sizes increased by about one percent per added target. Moreover, we found a significant negative slope for task experience (*B* = -0.70, *SE* = 0.32, *p* = .04), indicating that participants’ pupil sizes decreased with increasing experience by about 0.7% per day.

**Fig 4:**
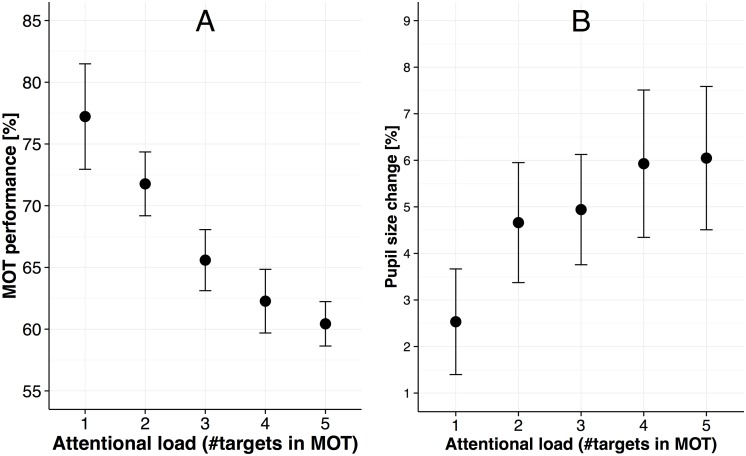
Control experiment results. (A) MOT task performance (i.e., fraction of correctly selected targets) as a function of attentional load and task experience. (B) Pupil size change relative to the passive viewing condition as a function of attentional load. Error bars in all panels are standard error of the mean.

As a point of note, with regard to the finding that pupil sizes systematically scale with attentional load in the MOT task, one might argue that increases in pupil sizes could be simply due to attending increasingly more dark edges with increasing targets in the MOT task and not due to increases in attentional load [13, 17, 45]. We investigated whether this potential confound applies to our data by conducting a control experiment in which targets with bright edges were tracked on a dark background with a separate set of participants (N = 20, 16 female, *M* = 21.70 years, *SD* = 3.31 years) that were measured for only one day. Except for changing the color of the background and edges of the targets, the experiment was exactly the same (see Fig 4A and 4B for the MOT performance and pupil sizes). For this set of participants, we again found that adding attentional load as a predictor to the baseline model significantly explained more variance in the data (*χ*^2^(2) = 22.20, *p* < .001, BIC = 516.4, baseline model’s BIC: 529.4). We then assessed the regression coefficient and it’s significance for this model and again found a significant positive slope for attentional load (*B* = 0.87, SE = 0.22, *p* = .001), suggesting that also for this control experiment participants’ pupil sizes increased by about one percent per added target.

Regarding the MOT performance of the control experiment, we also replicated the results of the main experiment. That is, when adding the predictor attentional load as a fixed and random effect to a baseline model, we found that these additions significantly explain additional variance in the data (*χ*^2^(2) = 26.14 *p* < .001, BIC = 739.5, baseline model’s BIC: 756.5). Assessing the regression coefficient for the predictor attentional load again yielded a significant negative slope of similar magnitude as in the main experiment (*B* = -4.14, SE = 0.78, *p* < .001), suggesting that for the control experiment the attentional load manipulation also had the desired effect.

As a next step, we compared whether pupil size changes between tracking one target and the passive viewing condition (i.e., the difference between performing the MOT and no task at all) differ from the changes between tracking conditions (e.g., the difference between tracking one and two targets). For this purpose, we extracted the fitted slopes (i.e., a slope is fitted for each participant separately) from the final model (i.e., the model including attentional load and task experience as predictors) and the intercepts for each participant. We compared the extracted slopes and intercepts using a paired t-test. Note, the fitted intercepts in this model represent the difference between the attentional load condition with one target and the passive viewing condition while the slopes represent the average increase in pupil sizes when the attentional load is increased by one unit, i.e., one target. We found that intercepts are significantly larger than slopes (mean difference: 1.85%, *t*(19) = 2.78, *p* = .012), suggesting that the difference in pupil sizes between the passive viewing condition and tracking one target condition is about 70% larger than the average difference between tracking conditions. We also tested whether this effect persists when we restricted the data to attentional load conditions one to four, one to three, and one to two, respectively. While on a descriptive level the direction of the effect was still present, we found that this effect was considerably smaller and no longer significant for these subsets of the data (one to two: 1.00% difference, *t*(19) = 1.56, *p* = .135; one to three: 0.43% difference, *t*(19) = 0.86, *p* = .403; one to four: 1.12%, *t*(19) = 1.96, *p* = .065). These findings suggest that the effect above for all attentional load conditions is potentially modulated by a ceiling effect (i.e., for each increase in attentional load the pupil sizes tend to become smaller).

## Discussion

In line with the results of the study by Alnæs and colleagues [16], we found that pupil sizes and tracking performance were systematically influenced by attentional load in a MOT task (i.e., the number of tracked targets). These findings suggest that pupil sizes reflect the current deployment of attentional resources to a task also on a finer scale as each increase in attentional load was accompanied by an increase in pupil sizes.

What is more, we found that repeating the experiment on two consecutive days led to a significant decrease in pupil sizes independent of the number of tracked targets in the MOT task. Moreover, these pupil size modulations over days were accompanied by slight performance improvements. Relatedly, previous work has shown that repeated presentations of a stimulus does lead to a pupil size decrease in a memory task [27], indicating a decrease in mental effort to retrieve the viewed stimulus from memory. Here, we show for the first time that performing an attentionally demanding task such as the MOT task on three consecutive days similarly led to not only an increase in performance, as might be expected, but also a concomitant decrease in pupil size as well. This reduction in pupil sizes over several days suggests a reduction of the attentional demand to perform the MOT task related to having extended experience with performing the task.

More generally, our findings suggest an interdependency between the performance in the MOT task and pupil sizes. Such an interplay may explain additional variance in the data beyond the variance explained by the attentional load and the task experience. We further investigated this observation by testing all possible models involving attentional load, task experience, and task performance as predictors. We found that the performance in the MOT task did not substantially explain additional variance on top of what was already explained by the number of tracked targets and days of measurements. Contrasting earlier findings that suggested that pupil size differences also reflect interindividual differences in information processing [46, 47], these findings suggest that changes in pupil sizes are sufficiently explained by the level of attentional load and task experience.

In addition, we found a higher pupil size increase for the comparison involving performing no task and tracking only one target than for the average pupil size increases for each additionally tracked target. This result suggests that pupil sizes not only reflect the current deployment of attentional resources related to attentional load but additionally reflect whether subjects are engaged in a task or not. However, we also found that this effect was considerably reduced in size and no longer significant when we restricted our analysis to a smaller set of attentional load conditions. Such a finding suggests that this effect may be modulated by a ceiling effect. That is, with increasing number of targets in the MOT task, the pupil size increases between attentional load conditions become systematically smaller. To resolve these issues, future studies could investigate attentional load conditions that are not prone to ceiling effects with a higher statistical power.

In conclusion, we find that pupil sizes are modulated by attentional load on a fine scale, i.e. each increment in attentional load led to a further increase in pupil sizes. In addition, pupil sizes are additionally modulated by whether humans are actively engaged in a task or not. Furthermore, pupil size modulations are systematically reduced with increasing task experience and improving task performance, suggesting also a close link between task performance and pupil sizes on a group level. Yet, modeling individual participants’ pupil sizes task performance does not additionally explain variance on top of the variance explained by the manipulations of attentional load and task experience. In fact, a model incorporating only the two predictors attentional load and task experience is selected as the best. Overall, this study demonstrates that pupil size modulations are affected by a wide range of different factors related to performing a task. These factors range from whether humans are actually performing a task or not, changes in attentional load, and changes in task experience.

More generally, the present findings suggest that pupil sizes could provide a general metric to assess attentional load in attention demanding visuospatial tasks (e.g., in visual search [48, 49] or visuomotor tasks [50]) without necessarily taking the task performance into account. Findings could also be applicable to demanding real world tasks (e.g., air-traffic control, driving a car, or flying an airplane) to assess the current attentional load during task performance. In particular, given that pupil sizes are modulated by attentional load on such a fine scale consistently over days, critical levels of attentional load (i.e., task-relevant information is no longer effectively processed or neglected) could be detected during task performance and timely interventions could prevent potential accidents (also see [29, 51–55]).

## Methods

### Methods of data acquisition

#### Participants

All participants were students of the University of Osnabrück. 20 students (14 female, *M* = 23.03 years, *SD* = 3.34 years) participated in the main experiment. In a control experiment, a separate set of 20 students (16 female, *M* = 21.70 years, *SD* = 3.31 years) participated. The study was approved by the ethics committee of the University Osnabrück. We informed participants about their rights and all participants signed a written consent form. Participants either received a monetary reward or course credits for participation.

#### Experimental setup

Participants sat in a dark room in front of a computer screen (BenQ XL2420T, resolution 1920 × 1080, 120 Hz, subtending a visual field of 32.87 × 18.49 visual degrees) at a distance of 90 cm. Eye movements were recorded using an Eyelink II (binocular tracking, 500 Hz sampling rate). In order to calibrate eye position, we used a 5-point grid and repeated calibration until the mean error was below 0.7 degrees for both eyes. As pupil size detection procedure, we selected the “diameter” option.

#### Experimental procedure

In the experiment, participants were instructed to track a subset of randomly chosen objects (‘targets’) among eighteen randomly moving objects (for a task overview, see Fig 1). Objects were 1.06 visual degrees in width. The subset of randomly chosen targets was varied between zero (a “passive viewing” condition) up to a maximum of five targets. At the beginning of a trial, all objects were shown for two seconds. Then, a subset of objects turned gray for two seconds, indicating the targets and then reverted back to look like all the other objects. After an additional 500 ms, objects started to move and participants were instructed to track the targets. While objects were moving, they repelled each other and bounced off the screen borders. The objects’ movement direction and velocity was randomly chosen within each frame with a probability of 0.01. With a refresh rate of 100 Hz this resulted in an average velocity of 2.57 visual degrees per second [minimum 1.71, maximum 3.42]. Objects were moving for a total of eleven seconds. During objects’ motion, all objects looked the same. After objects’ motion seized, participants were instructed to select the targets with a computer mouse. We suspected that preparing for selecting a certain number of targets could systematically influence pupil sizes across attentional load conditions (see supporting information in [56]). Therefore, to keep this executive processing demand constant in all trials, participants were instructed to always select five objects regardless of the number of targets that they were required to track. Importantly, participants were instructed to always select those objects that they think are the targets first. Participants were also informed that only the targets that were selected first counted towards their performance and only the number of selections matching the number of targets (e.g., if participants need to track two targets, then only the first two target selections count). After five objects were selected, correctly selected objects were shown in green. Participants were instructed to fixate on the fixation point (0.15 visual degrees radius) in the center of the screen while the objects moved randomly across the screen. Before each trial, a drift correction was applied for which participants needed to fixate the fixation point and press the space key on the keyboard. To ensure that the perceptual load is kept constant, participants always saw a total of eighteen objects in each trial. Before the experiment started, participants were familiarized with the task with three training trials that always contained two, three and zero targets. In the experiment, participants completed a total of 120 trials. Trials were grouped in blocks of six trials. In each block, each number of targets (i.e., zero to five) was done once in a randomized order. Repetition of the same target number in consecutive trials was avoided. The complete experiment took approximately one hour per participant.

Given the relatively large amount of trials per day, we assessed whether any general fatigue effects occurred by correlating the MOT performance with the number of trials for each day and participant separately. Generally, correlations were small and positive (mean *r* = .02), suggesting that there were no fatigue effects. However, correlations were significantly different from zero for the first (mean *r* = .04, *t* = 2.30, *p* = .033) and second (mean *r* = .04, *t* = 2.16, *p* = .043) but not for the third day (mean *r* = .01, *t* = 0.39, *p* = .702) of the measurements. This suggests that small training effects also occurred within an experimental session for the first two days of the measurements.

### Methods of data analysis

We downsampled the eye tracking data to 100 Hz. Data points with physiologically unlikely pupil sizes (i.e., negative pupil sizes as produced by blinks) within a trial were removed for each eye separately. Moreover, for each eye, we removed outliers within a trial using boxplots and applied 3 times the interquartile range as a threshold. Trials in which participant’s gaze deviated from the fixation point in the center by more than three visual degrees on average were removed from the analysis (for the main experiment: total of 8.38% trials excluded, *M* = 7.62 visual degrees, *SD* = 5.05 visual degrees; for the control experiment: total of 8.32% trials excluded, *M* = 10.66 visual degrees, *SD* = 9.37 visual degrees). Finally, for each trial and for each eye, we took the median pupil size for the time interval between three and nine seconds of the tracking period (i.e., when objects randomly moved across the screen). We chose this time interval to avoid early sensory confounds (i.e., contrast changes due to seeing a variable number of targets prior to the onset of the object motion) within the first several seconds in a trial and late executive confounds (i.e., effects of preparing motor actions to select targets at the end of a trial). A similar time interval has also been used in the study by Alnæs and colleagues [16]. We chose the median as a measure of central tendency as it is more robust towards outliers in the data. Specifically, these outliers could be implausible data points that are not filtered out by the boxplots that might occur due to technical malfunctions or remaining data points that preceded or followed blinks.

For investigating pupil size changes, we averaged across the median pupil sizes of the left and right eye for each trial. For the purpose of normalizing the data relative to the passive condition, all trials were averaged for each participant separately for each number of targets in the MOT task and day. Resulting values for targets between one and five were normalized relative to the passive viewing condition. In particular, we calculated the percent increase in pupil sizes relative to the passive viewing condition by dividing the pupil sizes in the target conditions by the pupil sizes in the passive viewing condition. As the attentional load conditions were presented in a randomized order (see section “Experimental Procedure” above for more details) the normalization step was not reasonable to perform on the trial level. Therefore, we performed the normalization step on the aggregated data. Moreover, due to randomly interleaving attentional load conditions, potential accumulations of pupil size increases over trials were averaged out.

## Supporting Information

S1 Fig Figure of pupil size traces as a function of attentional load for each day condition. (TIFF)

S1 File Zip file containing aggregated datasets underlying Figs 2, 3 and 4. (ZIP)

S2 File Link to the raw MOT task performance and eyetracking data (osf.io/qtzjb). (PDF)
