# Supplementary figures and images for: Pupil Sizes Scale with Attentional Load and Task Experience in a Multiple Object Tracking Task
Source: PLoS One. 2016 Dec 15;11(12):e0168087. doi: 10.1371/journal.pone.0168087 (PMC5157994; doi:10.1371/journal.pone.0168087)

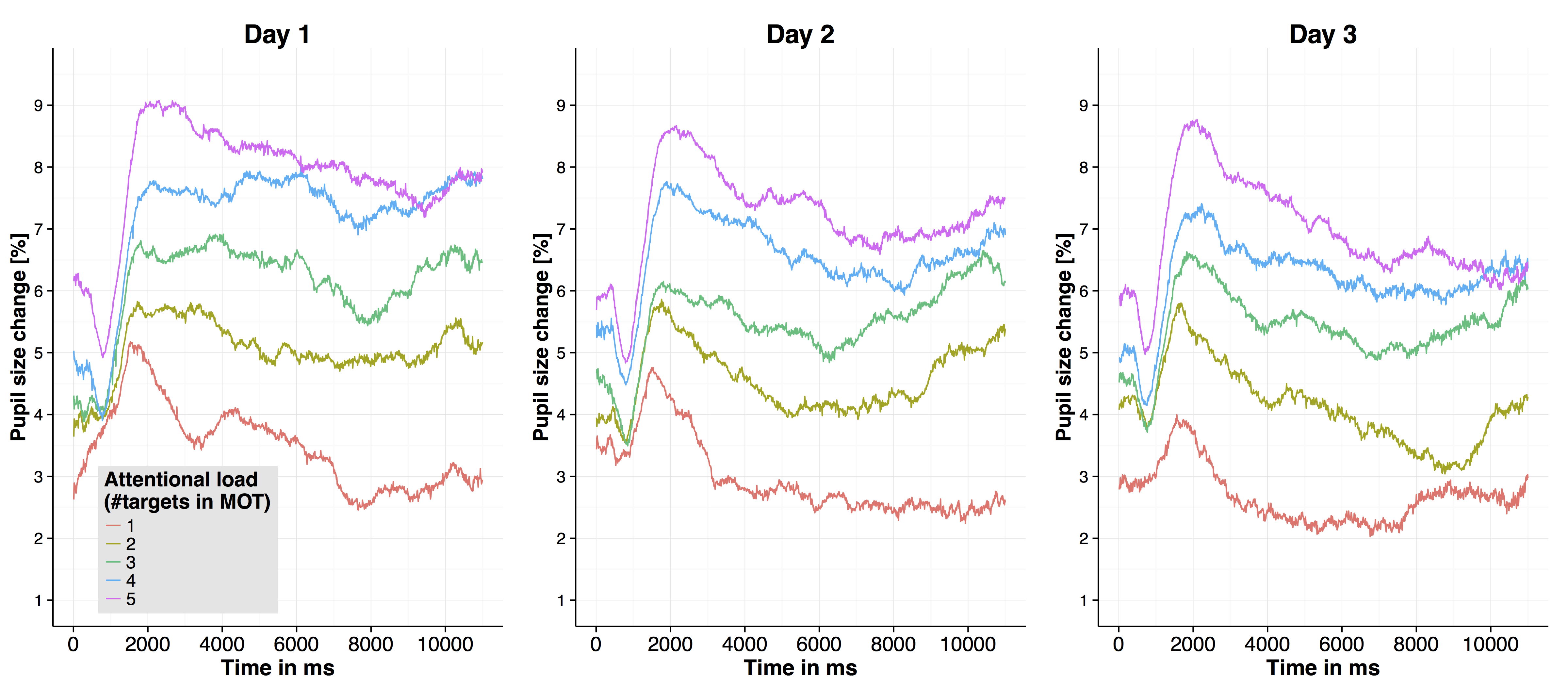

Supplement: S1 Fig — (TIFF) [file pone.0168087.s001.tiff]
