# Supplementary material for: Pupil Sizes Scale with Attentional Load and Task Experience in a Multiple Object Tracking Task
Source: PLoS One. 2016 Dec 15;11(12):e0168087. doi: 10.1371/journal.pone.0168087 (PMC5157994; doi:10.1371/journal.pone.0168087)
Supplement: S2 File — (PDF) [file pone.0168087.s003.pdf]

The raw MOT task performance and Eyetracking data can be accessed in the following repository: osf.io/qtzjb
